# Psychopathological Influences on Surgical and Clinical Outcomes in Lumbar Disk Herniation: Prediction Models and Literature Analysis

**DOI:** 10.3390/jpm15020048

**Published:** 2025-01-26

**Authors:** Gianluca Galieri, Vittorio Orlando, Edoardo Mazzucchi, Fabrizio Pignotti, Davide Cusumano, Paola Bazzu, Sofia Mantini, Roberto Altieri, Manlio Barbarisi, Alessandro Olivi, Giovanni Sabatino, Giuseppe La Rocca

**Affiliations:** 1 Institute of Neurosurgery, Fondazione Policlinico Universitario A. Gemelli IRCCS, Catholic University, 00168 Rome, Italy; vittorio.orlando01@icatt.it (V.O.); alessandro.olivi@policlinicogemelli.it (A.O.); giovanni.sabatino@policlinicogemelli.it (G.S.); giuseppe.larocca@policlinicogemelli.it (G.L.R.); 2 Neurosurgical Training Center and Brain Research, Mater Olbia Hospital, 07026 Olbia, Italy; edoardo.mazzucchi@ifo.it (E.M.); fabrizio.pignotti@materolbia.com (F.P.); 3 Department of Neurosurgery, IRCCS Regina Elena National Cancer Institute, 00144 Rome, Italy; 4 Department of Neurosurgery, Mater Olbia Hospital, 07026 Olbia, Italy; 5 Unit of Medical Physics, Mater Olbia Hospital, 07026 Olbia, Italy; davide.cusumano@materolbia.com; 6 Clinical Psychology Service, Mater Olbia Hospital, 07026 Olbia, Italy; paola.bazzu@materolbia.com; 7 Multidisciplinary Department, Charles University Faculty of Medicine in Pilsen, 323 00 Plzeň, Czech Republic; 22951053@cuni.cz; 8 Multidisciplinary Department of Medical-Surgical and Dental Specialties, University of Campania “Luigi Vanvitelli”, 81100 Caserta, Italy; roberto.altieri@unicampania.it (R.A.); manlio.barbarisi@unicampania.it (M.B.)

**Keywords:** lumbar disk herniation, psychological assessment, surgical outcomes, predictive modeling, preoperative evaluation

## Abstract

**Background/Objectives**: Lumbar disk herniation (LDH) significantly affects quality of life due to lower back and radiating leg pain. Surgical intervention, such as discectomy, is effective for symptom relief when conservative measures fail; however, psychological factors like anxiety, depression, and maladaptive coping strategies may negatively impact surgical outcomes. This study aims to assess the role of preoperative psychological evaluations in predicting postoperative recovery and to identify key psychological and functional predictors of surgical success. **Methods**: A prospective study was conducted on 888 patients undergoing microdiscectomy for LDH at Mater Olbia Hospital between December 2020 and December 2023. Preoperative evaluations included the Visual Analog Scale, Symptom Checklist 90-R, Oswestry Disability Index, and Short Form 36. Logistic regression models and ROC curve analysis were used to identify significant predictors of outcomes and evaluate model accuracy. **Results:** Preoperative pain levels and emotional well-being emerged as the strongest determinants of postoperative improvement in the Oswestry Disability Index. The predictive model demonstrated high specificity (90.2%) in identifying patients likely to benefit from surgery. Clinically significant improvements were achieved by 69% of patients, highlighting the importance of psychological and functional assessments. **Conclusions**: Preoperative psychological assessment is critical in predicting outcomes of lumbar disk herniation surgery. Addressing psychological factors preoperatively enhances recovery, supports personalized treatment planning, and improves patient education. These findings advocate for an integrated care model that considers both physical and psychological health, optimizing surgical outcomes and patient satisfaction.

## 1. Introduction

Lumbar disk herniation (LDH) is a prevalent condition, affecting approximately 1% to 3% of the population annually, with an overall prevalence of around 12%. It often leads to significant lower back pain that radiates into the lower limbs, and around 80% of individuals experience lower back pain at some point in their lives [1]. This radiating pain results from the rupture of the lumbar disk and the protrusion of the nucleus pulposus into the spinal canal, compressing the spinal nerves, which in turn contributes to impairment in daily activities and a substantial reduction in quality of life. When conservative treatments, such as physical therapy and medication, fail to provide relief, surgical intervention, such as microdiscectomy, is typically recommended. Microdiscectomy represents a type of spinal decompression surgery and it aims to alleviate pain, restore mobility, and improve overall function [2,3].

The complexities of pain in LDH are not solely anatomical but arise from a convergence of nociceptive, neuropathic, psychological, and social factors. For instance, psychological elements like maladaptive coping strategies, anxiety, and depression are increasingly recognized as key contributors to the persistence of pain and disability post-surgery. Several studies highlight that patients with preoperative anxiety or depression face a higher likelihood of postoperative complications, longer hospital stays, and increased rates of hospital readmission. Such psychological states can amplify pain perception and contribute to a cycle of disability and fear of movement, known as kinesiophobia, which exacerbates the overall outcome [4,5,6,7].

Furthermore, maladaptive beliefs, such as fear of movement and pain catastrophizing, have been identified as predictors of both preoperative and postoperative function in spine surgery patients, showing a direct impact on recovery trajectories [8]. This biopsychosocial model of pain is supported by research indicating that factors like low work satisfaction, prolonged sick leave, and poor expectations regarding return to work can serve as risk factors for ongoing pain and disability in lumbar disk surgery patients [9,10]. While these studies often focus on degenerative lumbar conditions, the implications are relevant to LDH, where psychological components can create a feedback loop of pain and functional limitation.

Interestingly, the mental health impact of chronic radicular pain can itself become a cause of depression and anxiety, creating a complex interplay between physical disability and psychological distress [11,12]. This underscores the need for a comprehensive, preoperative psychological evaluation in patients undergoing surgery for LDH. By assessing factors like fear-avoidance beliefs, coping mechanisms, and psychological readiness, healthcare providers may identify individuals at higher risk for unsatisfactory outcomes and implement preoperative or postoperative interventions to enhance recovery. Evidence suggests that interventions such as patient education programs can effectively reduce anxiety levels in patients awaiting spine surgery, potentially improving postoperative outcomes [13,14].

The objective of this study is to evaluate whether preoperative psychological assessment can serve as a predictor of postoperative outcomes in patients undergoing surgery for lumbar disk herniation. This research aims to highlight the impact of preoperative psychological factors on both short- and long-term recovery, advocating for an integrated approach to patient care that addresses both physical and psychological dimensions of recovery.

## 2. Materials and Methods

### 2.1. Patient Selection

A prospective study was conducted on 888 patients with symptomatic LDH who underwent surgery between December 2020 and December 2023 at the Neurosurgery Department of Mater Olbia Hospital. All patients provided written informed consent, and the study received prior approval from the Ethics Committee of Regione Autonoma Sardegna (protocol number 276/2020/CE of 29 October 2020). Patients were followed for at least 1 year postoperatively to monitor outcomes.

Inclusion criteria include the following:

- Age between 18 and 80 years
- Symptomatic LDH for more than 6 weeks, unresponsive to conservative treatments (e.g., physical therapy, pain management)
- No signs of lumbar spinal stenosis or instability
- Minimum follow-up of 1 year
- LDH localized between spinal levels L1 and S1
- Consent to participate in the study

Exclusion criteria include the following:

- Previous or current treatment for anxiety or depression
- Psychotherapy in the last 2 years
- History of neurotoxic chemotherapy
- Other sources of chronic pain, such as osteoporosis
- Active neoplastic disease or cognitive impairment

Withdrawal criteria include the following:

- Loss to follow-up
- Unrelated medical conditions (e.g., neoplastic diseases or systemic infections that could interfere with surgical outcome interpretation
- Withdrawal of informed consent
- Non-related death

### 2.2. Psychological and Functional Disability Assessment

Patients with surgical indications for LDH who agreed to participate underwent a psychological and functional impairment assessment before surgery. The following validated scales were administered preoperatively to evaluate pain, psychological status, and functional disability:

Visual Analog Scale (VAS): Both for Back Pain (VAS-BP) and Leg Pain (VAS-LP), on a scale from 0 to 10, where a lower score indicates less pain [15]. It has been validated for its reliability and sensitivity in detecting changes in pain levels in patients undergoing spinal surgery [16].

Symptom Checklist 90-R (SCL90-R): A 90-item self-reported questionnaire assessing psychological symptoms, categorized into nine subscales: Somatization, Obsessivity, Interpersonal Sensitivity, Depression, Anxiety, Hostility, Phobic Anxiety, Paranoid Ideation, and Psychoticism [17,18]. The SCL-90-R has demonstrated strong internal consistency (Cronbach’s alpha > 0.75) and validity in both clinical and non-clinical populations, including those with chronic pain [19].

Oswestry Disability Index (ODI): The ODI is a gold-standard measure of functional disability specific to lower back conditions. It has been validated with excellent test-retest reliability (intraclass correlation coefficient > 0.90) and internal consistency (Cronbach’s alpha > 0.85). It is widely used in lumbar spine surgery to assess the degree of disability and monitor recovery [20].

Short Form 36 (SF-36): The SF-36 measures health-related quality of life across eight domains, including physical functioning, mental health, and pain. It has been extensively validated in clinical research with high reliability (Cronbach’s alpha > 0.70) and sensitivity to changes in health status over time [21,22].

EuroQoL-5 Dimensions (EQ-5D): The EQ-5D is a standardized instrument assessing health-related quality of life based on five dimensions: mobility, self-care, usual activities, pain/discomfort, and anxiety/depression. It has been validated in diverse patient populations and is considered a reliable measure for health outcomes in spinal disorders [23].

All scales, except for the SCL90-R, were re-administered during follow-up assessments. The SCL90-R is structured to capture symptoms experienced during the prior week, with nine subscales used to define psychopathological dimensions and three global indices: the Global Severity Index (GSI), indicating overall psychological distress; the Positive Symptom Distress Index (PSDI), measuring symptom intensity; and the Positive Symptom Total (PST), representing the number of reported symptoms. Additionally, a Current Symptom Index (CSI) was recorded, and calculated as the mean value of Somatization, Obsessivity, Depression, Anxiety, Phobic Anxiety, and Psychoticism [24]. Since a pathological cutoff for spine surgery patients has not been defined, raw scores were used in the analysis.

### 2.3. Surgical Treatment

All surgeries were conducted by a consistent team of two experienced surgeons to ensure uniformity in the surgical approach and technique for lumbar discectomy. Discectomy was selected as the surgical intervention for these patients, with the primary goal of removing herniated disk material to decompress the affected nerve root, alleviate pain, and restore function in patients with LDH. For detailed surgical techniques, we referred to the methodology described by La Rocca et al. [25].

### 2.4. Statistical Analysis

The predictive ability of clinical and psychological factors for determining clinical outcomes was evaluated using univariate analysis.

Three clinical outcomes were considered: variation in ODI, VAS leg pain, and VAS back pain. Depending on the normality of the data distribution relative to the studied outcome, either the Wilcoxon-Mann Whitney test or the t-test was applied, with normality assessed using the Shapiro-Wilk test [26,27]. To address the issue of multiple comparisons, the Benjamini-Hochberg procedure was employed to adjust the *p*-values obtained from the Wilcoxon test [28].

A cross-correlation matrix was constructed for variables that demonstrated significance in the univariate analysis, using Pearson’s correlation coefficient (PCC) as the correlation metric [29]. Variables with PCC values below |0.5| were considered uncorrelated. The most significant variable from the univariate analysis was then used to develop a logistic regression model [30]. The model’s Receiver Operating Characteristic (ROC) curve was calculated, and the area under the curve (AUC) was determined using a bootstrap method with 2000 samples to calculate the 95% confidence interval [31]. The optimal cut-off point was identified through the Youden Index, with sensitivity, specificity, and both negative and positive predictive values calculated at that threshold.

All statistical analyses and data processing were conducted using R software (version 4.3.3) and its dedicated packages (R Core Team, Vienna, Austria) [32,33].

## 3. Results

Between December 2020 and December 2023, a total of 952 patients with LDH underwent surgical intervention at Mater Olbia Hospital. Of these, 64 patients were lost to follow-up and subsequently excluded from the study. Therefore, the analysis included 888 patients with complete data.

### 3.1. Patient Demographics and Clinical Characteristics

The cohort consisted of 522 males and 366 females, with a mean age of 53.34 years (SD = 12.81). The average body mass index (BMI) was 24.9 (SD = 3.98). In terms of employment status, 560 patients were employed, while 328 were unemployed. Among the patients, 356 had a diagnosis of arterial hypertension, 101 had an autoimmune rheumatologic disease, and 384 were smokers (Table 1).

### 3.2. Surgical Data

The total number of herniated disks operated on in this cohort was 905, as some patients underwent surgery at multiple levels. Specifically, 859 patients had single-level disk herniations, while 23 had two-level herniations. The distribution of surgical levels was as follows: 348 herniations at the L5-S1 level, 398 at L4-L5, 110 at L3-L4, 46 at L2-L3, and 3 at L1-L2. The mean surgical incision length was 3.41 cm (SD = 0.39), and no patients required subcutaneous drainage postoperatively (Table 2).

### 3.3. Hospitalization Data

The mean length of hospital stay was 2.25 days (SD = 0.55), and the mean operative time was 48 min (SD = 13). Patients were followed for an average of 12 to 48 months postoperatively (Table 2).

### 3.4. Complications

Intraoperative complications occurred in 22 cases, of which 19 were due to dural tears, 1 involved a vertebral fracture, and 2 involved a subcutaneous hematoma. Postoperative complications were reported in 46 cases, including 21 instances of recurrent disk herniation, 22 cases of stenosis-related instability, 1 superficial wound infection, and 2 cases of persistent sensory deficit in the lower limb (Table 3).

### 3.5. Pre- and Postoperative Clinical Outcomes

The patients showed significant improvements in all measured clinical parameters post-surgery. The Oswestry Disability Index (ODI) decreased from a preoperative mean of 55.27% (SD = 18.93) to a postoperative mean of 20.14% (SD = 16.44). The Visual Analog Scale (VAS) scores for leg pain decreased from a mean of 8.32 (SD = 1.32) preoperatively to 1.80 (SD = 1.50) postoperatively, while the VAS scores for back pain reduced from 7.90 (SD = 1.30) preoperatively to 2.46 (SD = 1.44) postoperatively. The EQ-5D health-related quality of life score improved from a mean of 0.316 (SD = 0.19) preoperatively to 0.721 (SD = 0.43) postoperatively (Table 4).

**Figure 1:**
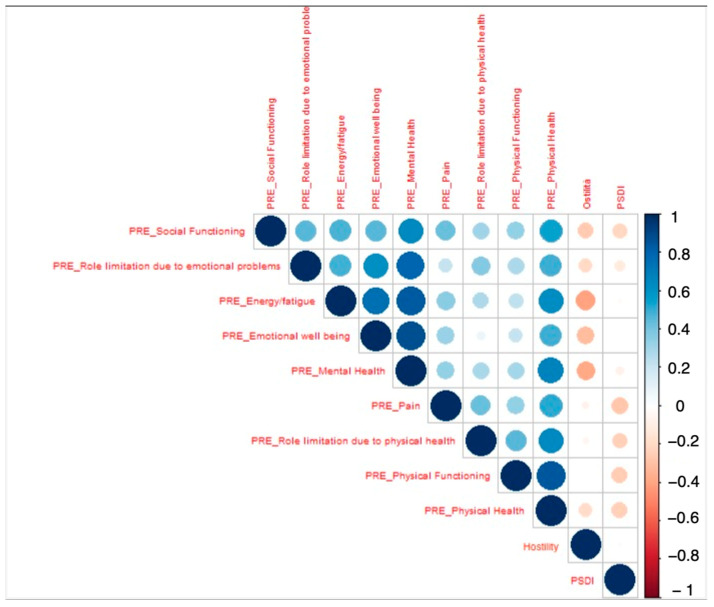
Cross-correlation matrix displaying the relationships among statistically significant features from univariate analysis of the difference in pre- and postoperative ODI with a threshold of 20 points. PSDI: Positive Symptom Distress Index.

The primary outcome was the ODI, which measures disability related to lumbar spine pathology. A threshold of 20 points was used to define clinically significant improvement, representing the difference between pre- and postoperative scores. Overall, 613 of 888 patients (69%) achieved an improvement of 20 points or more, indicating a favorable surgical outcome. Conversely, 275 of 888 patients (31%) showed a minimal improvement of less than 20 points. Statistical analysis identified several preoperative variables significantly associated with postoperative improvement. After adjusting for multiple comparisons using the Benjamini-Hochberg correction, the following predictors remained significant (Table 5):

The correlation matrix (Figure 1) illustrates the relationships between the preoperative variables. Significant correlations were observed between preoperative pain and physical functioning, as well as between mental health and social functioning. These findings suggest that preoperative psychological and physical health are interdependent factors influencing surgical outcomes.

A predictive model was then elaborated combining the level of pain before surgery and the level of hostility.

The mathematical formulation of the predictive model was the follows:

$$ln\left[\frac{p\left(x\right)}{1-p\left(x\right)}\right]=ax+by+c$$

where *p*(*x*) was the probability of ODI improvement, *x* was the level of pain before surgery, and *y* was the level of hostility. As the coefficients, *a* was equal to −0.064 ± 0.001, *b* was −0.228 ± 0.229, and *c* was 1.288 ± 0.167.

**Figure 2:**
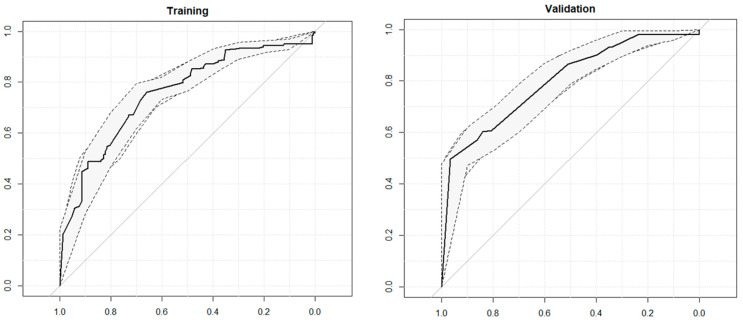
ROC curves for training and validation predictive model of the difference in pre- and postoperative ODI with a threshold of 20 points. The ROC curves illustrate the model’s predictive performance, with the x-axis representing specificity and the y-axis representing sensitivity. The solid black line corresponds to the ROC curve, while the dashed lines indicate the 95% confidence interval. The diagonal gray line represents the performance of a random classifier.

The predictive model showed strong discrimination in identifying patients likely to achieve significant improvement. The Area Under the Curve (AUC) was 0.74 (95% CI: 0.70–0.78) in the training set and 0.80 (95% CI: 0.75–0.85) in the validation set (Figure 2). At the optimal threshold (0.82), the model achieved a specificity of 90.2% and a sensitivity of 46.7%. These results indicate that the model is highly specific in identifying patients with favorable outcomes, although sensitivity remains moderate with AUC values that can be considered acceptable [34] (Figure 2).

A secondary analysis was conducted on the improvement in VAS Back Pain scores, setting a clinical improvement threshold at a 25% reduction in pain scores post-surgery. An outcome rate of 25% was observed, indicating that a quarter of the patient population did not achieve this level of pain reduction, underscoring the challenge in managing back pain even post-surgically. Through the rigorous statistical analysis, several preoperative psychosomatic features were identified as significant predictors of less-than-expected improvement in VAS Back Pain scores. These features and their corresponding *p*-values, adjusted for multiple testing, are detailed in Table 6.

**Figure 3:**
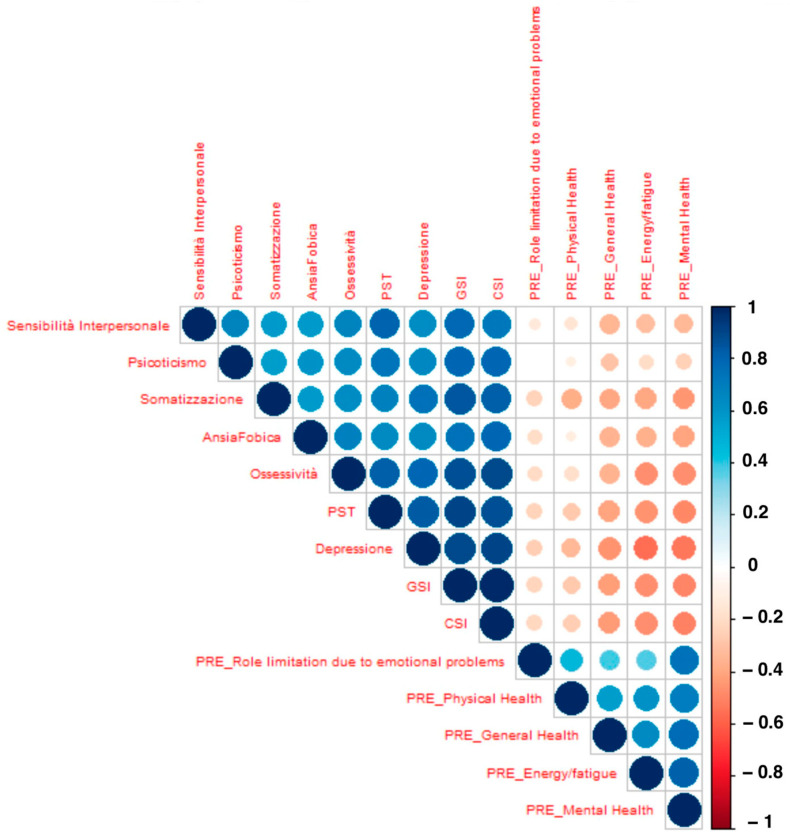
Cross-correlation matrix showing the relationships among statistically significant features from univariate analysis of the difference in pre- and postoperative VAS back pain with a threshold of 25%.

Analysis of the correlation matrix revealed significant interrelationships among preoperative psychosomatic variables (Figure 3). All the significant features showed a high correlation with the most significant feature (somatization), so the predictive model was elaborated considering only the somatization level as a single variable.

$$ln\left[\frac{p\left(x\right)}{1-p\left(x\right)}\right]=ax+b$$

In this case, *p*(*x*) was the probability of VAS Back pain improvement, *x* was the level of somatization, *a* was equal to −2.231 ± 0.591, and *b* was 3.778 ± 0.853.

Notable correlations included the following:

- Somatization and GSI: High correlation, indicating that patients with extensive somatic symptoms often exhibit severe psychological distress.
- Depression and CSI: A strong link, suggesting a substantial impact of depressive states on cognitive perceptions of health and illness.
- Interpersonal Sensitivity and Role Limitation due to Emotional Problems: Moderate to high correlation, impacting social interactions and psychological well-being.

**Figure 4:**
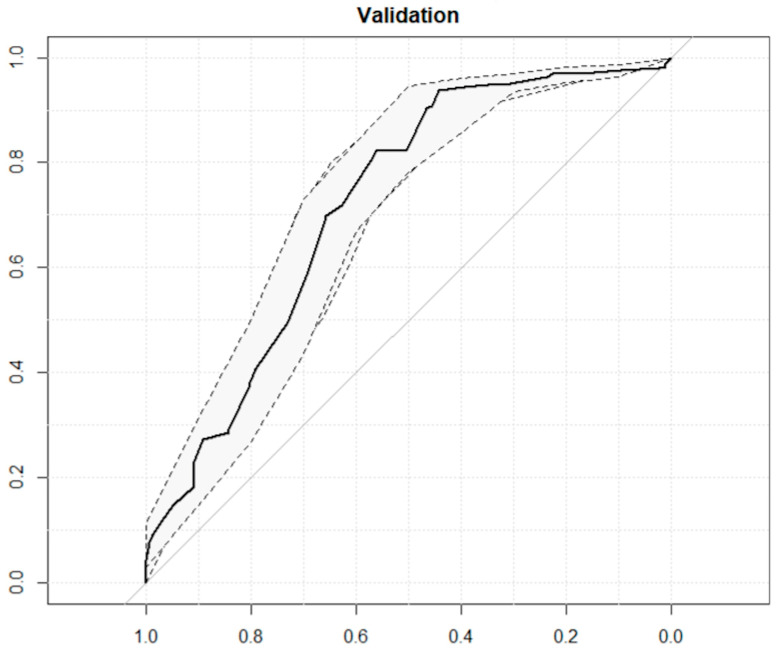
ROC curves for validation predictive model of the difference in pre- and postoperative VAS back pain with a threshold of 25% training (left) and validation (right). The ROC curves illustrate the model’s predictive performance, with the x-axis representing specificity (0.72) and the y-axis representing sensitivity. The solid black line corresponds to the ROC curve, while the dashed lines indicate the 95% confidence interval. The diagonal gray line represents the performance of a random classifier.

The ROC curve (Figure 4) analysis evaluated the discriminatory power of the model based on the following psychosomatic predictors:

- Training Set: Achieved an AUC of 79.9% (CI: 69.2–90.6) with a sensitivity of 85.7% and specificity of 69.0%. The optimal threshold was identified at 0.5799.
- Validation Set: Demonstrated an AUC of 71.9% (CI: 67.2–76.5) with a sensitivity of 82.2% and specificity of 56.2%. The threshold was set at 0.6568 (Table 7).

As for VAS leg pain, an improvement was visible in 90% of the patients. No significant features were observed among the ones investigated in the study, so no predictive model was elaborated. This was probably related to the significant disproportion among the two classes of patients.

## 4. Discussion

In our exploration of psychological factors affecting outcomes in lumbar disc herniation surgery, a comprehensive narrative emerges from the synthesis of key findings across several studies. The interplay between mental health and surgical recovery is intricate, as demonstrated by the varied aspects of patient psychology discussed below.

The prevalence and impact of depression and anxiety in surgical patients were robustly demonstrated in a study that found significant levels of these conditions among lumbar disc herniation patients, with approximately one-third exhibiting clinical symptoms. These psychological states significantly compromised recovery, increasing the risk of poor surgical outcomes by up to 50% [35].

The decision to undergo surgery is profoundly influenced by a patient’s perceived quality of life and functional ability. Research has shown that patients with lower preoperative quality of life scores were more than twice as likely to express dissatisfaction with surgical outcomes [36].

Catastrophizing, or the tendency to envision the worst possible outcome, has been closely linked to increased postoperative pain and diminished quality of life. One study quantified this effect, noting that high catastrophizing scores were associated with a 40% increase in post-surgical pain perception [37].

The longitudinal influence of preoperative depression extends far beyond immediate postoperative periods, as demonstrated in a study that followed patients for over a year. Patients with moderate to severe depression were found to have three times the likelihood of experiencing suboptimal recovery outcomes [38].

The broader clinical implications of psychological well-being are illustrated by findings that link preoperative psychological health with long-term recovery success. Patients reporting better psychological states prior to surgery had a substantially higher probability of positive outcomes, with a 60% increase in long-term success rates [39].

Anxiety not only affects immediate postoperative pain management but also predicts longer-term analgesic usage. Patients with higher anxiety levels preoperatively were noted to require 25% more pain medication postoperatively [40].

Fear-avoidance beliefs are another psychological factor with a measurable impact on surgical outcomes. Patients who exhibited these beliefs were 1.5 times more likely to experience poor outcomes, emphasizing the need for interventions that address these fears through cognitive behavioral strategies, thereby potentially improving recovery experiences [41].

The necessity for tailored psychological interventions is further reinforced by findings that link trait anxiety with persistent pain issues. A significant association was found between high levels of anxiety and ongoing radicular pain a year after surgery, suggesting that patients with such traits might benefit from targeted psychological support [42,43].

The importance of integrating psychological assessments into early postoperative rehabilitation is supported by a study where patients participating in early active rehabilitation programs that included psychological assessments showed an 80% improvement in function and pain-related psychometric scores within 6 months [44].

Wang et al. identified several key factors that predict patient dissatisfaction 2 years post-discectomy for lumbar disc herniation among an older Chinese cohort. Over 70% of patients reported satisfaction with their discectomy for lumbar disc herniation. Key predictors of dissatisfaction, identifiable before surgery, include obesity and preoperative depression. Additionally, factors such as symptom recurrence and postoperative depression were linked to reduced patient satisfaction post-surgery [45].

In line with these studies, our results demonstrate that preoperative emotional well-being, as measured by the SCL-90-R and SF-36, is a strong determinant of postoperative improvement in the Oswestry Disability Index (ODI). Specifically, patients with better preoperative psychological profiles achieved clinically significant improvements (≥20-point ODI reduction) at a rate of 69%, underscoring the critical role of emotional health in recovery.

Patients with heightened attention to pain-related stimuli [46] and those exhibiting pain-avoidance behaviors [47] often experience worse functional outcomes postoperatively. Such behaviors perpetuate a cycle of physical inactivity, psychological distress, and delayed recovery. Conversely, patients with obsessive-compulsive traits may paradoxically preserve pain as a coping mechanism to manage underlying emotional distress [48,49].

Importantly, surgical intervention can yield significant improvements even in patients with preoperative psychological distress, such as depression. However, depression remains a critical factor that can impede recovery [50]. It is associated with increased postoperative complications, higher opioid consumption, prolonged hospital stays, and elevated healthcare costs [51]. Additionally, the immunosuppressive effects of psychological stress may increase susceptibility to infections and other complications, underscoring the need for perioperative psychological management [52].

Our findings diverge from some earlier studies in that somatization emerged as the most significant predictor of postoperative back pain persistence (VAS). This suggests that interventions targeting somatic symptom reduction, rather than solely focusing on anxiety or depression, may yield better pain management outcomes.

These findings underline the critical role of psychological factors in optimizing outcomes for patients undergoing lumbar disk herniation surgery, with implications for both surgeons and mental health care professionals. Integrating preoperative psychological assessments, such as the SCL-90-R and SF-36, into the standard surgical workflow can help identify patients at higher risk of suboptimal recovery. By leveraging these tools, surgeons gain insights into patients’ emotional and psychological readiness, allowing for the development of tailored interventions even before the surgery takes place. Predictive models, incorporating variables such as preoperative pain levels and hostility, not only support the stratification of patients but also enable more personalized surgical planning. This approach helps guide realistic discussions with patients regarding expected postoperative outcomes, ultimately improving satisfaction and adherence to recovery protocols.

For mental health care professionals, these results emphasize the importance of close collaboration with the surgical team. Psychological interventions, such as cognitive-behavioral therapy (CBT), psychoeducation, and stress management programs, should be considered essential components of care for patients presenting with high levels of anxiety, depression, or somatization. The significant impact of somatization on persistent postoperative back pain suggests the need for targeted strategies aimed at reducing somatic symptoms. Techniques focusing on mindfulness, resilience, and adaptive coping mechanisms may play a pivotal role in mitigating these challenges and enhancing recovery.

## 5. Strengths and Limitations

This study benefits from a large sample size, ensuring robust statistical power, and the use of validated tools (SCL-90-R, ODI, VAS, and EQ-5D) for comprehensive psychological and functional assessment. The consistent surgical approach minimizes variability, while advanced statistical methods strengthen the reliability of the findings.

This study has some limitations that should be acknowledged. First, being conducted in a single-center setting may limit the generalizability of the findings to other institutions. Additionally, the exclusion of patients with pre-existing psychological conditions may have introduced selection bias, underrepresenting individuals with significant psychological comorbidities.

The fact that all surgeries were conducted by only two surgeons may limit the generalizability of the findings, as surgical experience and techniques can vary significantly across surgeons.

The follow-up period, ranging from 12 to 48 months, does not capture long-term outcomes or delayed complications. Self-reported measures, while validated, may be prone to response bias, and the absence of established psychological cut-offs for spine surgery patients limits clinical applicability.

Statistically, while robust methods were used, the moderate sensitivity of the predictive model suggests room for improvement in identifying all patients with potential for significant recovery. Furthermore, unmeasured confounding factors, such as socioeconomic status and access to rehabilitation, may have influenced the results.

## 6. Conclusions

This study highlights the critical role of comprehensive psychological assessments in optimizing surgical outcomes for lumbar disk herniation. By addressing both physical and emotional dimensions of care, healthcare providers can adopt a patient-centered approach that enhances recovery, improves quality of life, and fosters long-term functional health. Integrating psychological evaluations into both pre- and postoperative workflows enables precise surgical planning, strengthens the doctor–patient relationship through transparent communication about outcomes, and supports multidisciplinary collaboration.

The findings underline the importance of tailored interventions targeting psychological factors such as somatization, anxiety, and depression, which significantly influence recovery trajectories. This integrated model of care not only ensures holistic recovery but also sets a foundation for further investigations into the cost-effectiveness and long-term benefits of multidisciplinary strategies. Future research should explore the development and validation of predictive models across diverse patient populations to refine personalized treatment pathways and improve patient satisfaction globally.

## Figures and Tables

**Table 1:** Demographic data.

Demographic Data	No of Patients
Number of patients	888
Average Age	53.34 years (SD: 12.81)
Male/Female Ratio	522/366
Mean BMI	24.9 (SD: 3.98)
Employed/non-employed	560/328
Smokers/Non-smokers	384/504
Arterial Hypertension	356
Previous lumbar surgery	132
Fibromyalgia	101
Mean Follow-up	12–48 months

**Table 2:** Surgical data and operated levels.

Lumbar Hernia Treated	No of Levels	Surgical Data	
Total lumbar hernia	905	Average wound size	3.41 cm (+/− 0.39)
Single level	859	Mean surgical time	48 min (+/− 13)
Double level	23	Average Hospital Stay	2.25 days (+/− 0.55)
L5S1	348		
L4L5	398		
L3L4	110		
L2L3	46		
L2L1	3		

**Table 3:** Intraoperative and postoperative complications.

Intraoperative Complications	No of Complications	%	Postoperative Complications	No of Complications	%
Dural tear	19	2.14	Recurrence	21	2.36
Vertebral fracture	1	0.11	Instability	22	2.48
Subcutaneous hematoma	2	0.23	Motor deficit	0	0
			Sensory deficit	2	0.23
			Wound infection	1	0.11

**Table 4:** Clinical outcome with preoperative and postoperative assessment.

Patient Assessment	Preoperative	Postoperative	*p*-Value
ODI (Oswestry Disability Index)	55.27% (+/− 18.93)	20.14% (+/− 16.44)	<0.001
VAS (Visual Analog Scale) Leg Pain	8.32 (+/− 1.32)	1.80 (+/− 1.50)	<0.001
VAS (Visual Analog Scale) Back Pain	7.90 (+/− 1.30)	2.46 (+/− 1.44)	<0.001
EQ-5D (Euro Quality of life—5 Dimensions)	0.316 (+/− 0.19)	0.721 (+/− 0.43)	<0.001

**Table 5:** Significant features in the univariate analysis of the difference in pre- and postoperative ODI with a threshold of 20 points.

Features	*p*-Value	*p*-Value Adjusted
Pain pre-op	<0.001	<0.001
Emotional well-being pre-op	<0.001	<0.001
Social Functioning pre-op	<0.001	<0.001
Mental Health pre-op	<0.001	<0.001
Role limitation due to emotional problems pre-op	<0.001	<0.001
Physical Health pre-op	<0.001	<0.001
Role limitation due to physical health pre-op	<0.002	<0.001
Energy/Fatigue pre-op	<0.003	<0.014
PSDI	<0.005	<0.019
Hostility	<0.003	<0.014
Physical Functioning pre-op	<0.005	<0.019

**Table 6:** Significant features in the univariate analysis of the difference in pre- and postoperative VAS back pain with a threshold of 25%.

Features	*p*-Value	*p*-Value Adjusted
Somatization	<0.001	<0.001
Depression	<0.001	<0.001
CSI	<0.001	<0.001
GSI	<0.001	<0.001
Mental Health pre-op	<0.001	<0.003
Energy/Fatigue pre-op	<0.001	<0.003
General Health pre-op	<0.002	<0.008
Physical Health pre-op	<0.002	<0.008
PST	<0.002	<0.008
Obsessivity	<0.002	<0.008
Interpersonal Sensitivity	<0.004	<0.017
Role limitation due to emotional problems pre-op	<0.052	<0.017
Phobic Anxiety	<0.005	<0.017
Psychoticism	<0.006	<0.019

**Table 7:** Predictive performance of the logistic model for training and validation analysis of the difference in pre- and postoperative VAS back pain with a threshold of 25%.

	Sensitivity	Specificity	Threshold	J-Index	AUC	Low AUC	High AUC
Training	85.7	68.9	0.57	0.54	79.92	69.23	90.62
Validation	82.1	56.1	0.65	0.38	71.87	67.22	76.52

## Data Availability

The data presented in this study are available on request from the corresponding author.

## Abbreviations

The following abbreviations are used in this manuscript:

AUC	Area Under the Curve
BMI	Body Mass Index
CSI	Current Symptom Index
EQ-5D	EuroQol-5 Dimensions
GSI	Global Severity Index
LDH	Lumbar Disk Herniation
ODI	Oswestry Disability Index
PCC	Pearson’s Correlation Coefficient
PSDI	Positive Symptom Distress Index
PST	Positive Symptom Total
ROC	Receiver Operating Characteristic
SCL-90-R	Symptom Checklist 90-Revised
SD	Standard Deviation
SF-36	Short Form 36
VAS	Visual Analog Scale
VAS-BP	Visual Analog Scale for Back Pain
VAS-LP	Visual Analog Scale for Leg Pain
